# Cost-effectiveness of maternal GBS immunization in low-income sub-Saharan Africa

**DOI:** 10.1016/j.vaccine.2017.07.108

**Published:** 2017-12-14

**Authors:** Louise B. Russell, Sun-Young Kim, Ben Cosgriff, Sri Ram Pentakota, Stephanie J. Schrag, Ajoke Sobanjo-ter Meulen, Jennifer R. Verani, Anushua Sinha

**Affiliations:** a Institute for Health and Department of Economics, Rutgers University, New Brunswick, NJ, USA; b Department of Healthcare Management and Policy, School of Public Health, Seoul National University, 1 Gwanak-ro, Gwanak-gu, Seoul 08826, South Korea; c Westfield, NJ, USA; d Department of Surgery, New Jersey Medical School, Rutgers University, Newark, NJ, USA; e National Center for Immunization and Respiratory Diseases, Centers for Disease Control and Prevention, 1600 Clifton Rd, Atlanta, GA 30333, USA; f Bill & Melinda Gates Foundation, 1432 Elliott Ave, Seattle, WA 98119, USA; g Department of Health Systems and Policy, School of Public Health, Rutgers University, Piscataway, NJ, USA

**Keywords:** Group B streptococcus (GBS), Neonatal sepsis, Maternal GBS vaccine, Cost-effectiveness, Low-income sub-Saharan Africa, ANC1, proportion of women with at least 1 antenatal visit, ANC4, proportion of women with 4 or more antenatal visits, CFR, case fatality ratio, CEA, cost-effectiveness analysis, DALY, disability-adjusted life-year, EOGBS, early-onset GBS disease, GAVI, Global Alliance for Vaccines and Immunization, GBS, Group B streptococcus, GDP, gross domestic product, GDPpc, gross domestic product per capita, HIV, human immunodeficiency virus, LMICs, low- and middle-income countries, LOGBS, late-onset GBS disease, PSA, probabilistic sensitivity analysis, WHO, World Health Organization, WHO-CHOICE, WHO CHOosing Interventions that are Cost-Effective

## Abstract

**Background:**

A maternal group B streptococcal (GBS) vaccine could prevent neonatal sepsis and meningitis. Its cost-effectiveness in low-income sub-Saharan Africa, a high burden region, is unknown.

**Methods:**

We used a decision tree model, with Markov nodes to project infants’ lifetimes, to compare maternal immunization delivered through routine antenatal care with no immunization. 37 countries were clustered on the basis of economic and health resources and past public health performance. Vaccine efficacy for covered serotypes was ranged from 50% to 90%. The model projected EOGBS (early-onset) and LOGBS (late-onset) cases and deaths, disability-adjusted life years (DALYs), healthcare costs (2014 US$), and cost-effectiveness for a representative country in each of the four clusters: Guinea-Bissau, Uganda, Nigeria, and Ghana. Maximum vaccination costs/dose were estimated to meet two cost-effectiveness benchmarks, 0.5 GDP and GDP per capita/DALY, for ranges of disease incidence (reported and adjusted for under-reporting) and vaccine efficacy.

**Results:**

At coverage equal to the proportion of pregnant women with ≥ 4 antenatal visits (ANC4) and serotype-specific vaccine efficacy of 70%, maternal GBS immunization would prevent one-third of GBS cases and deaths in Uganda and Nigeria, where ANC4 is 50%, 42–43% in Guinea-Bissau (ANC4 = 65%), and 55–57% in Ghana (ANC4 = 87%). At a vaccination cost of $7/dose, maternal immunization would cost $320-$350/DALY averted in Guinea-Bissau, Nigeria, and Ghana, less than half these countries’ GDP per capita. In Uganda, which has the lowest case fatality ratios, the cost would be $573/DALY. If the vaccine prevents a small proportion of stillbirths, it would be even more cost-effective. Vaccination cost/dose, disease incidence, and case fatality were key drivers of cost/DALY in sensitivity analyses.

**Conclusion:**

Maternal GBS immunization could be a cost-effective intervention in low-income sub-Saharan Africa, with cost-effectiveness ratios similar to other recently introduced vaccines. The vaccination cost at which introduction is cost-effective depends on disease incidence and vaccine efficacy.

Clinical Trial registry name and registration number: Not applicable.

## Highlights

• Maternal GBS vaccination could prevent many neonatal deaths in low-income sub-Saharan Africa.
• Immunization during pregnancy could cut GBS deaths by 30%-55% in typical sub-Saharan settings.
• To show the full cost of vaccination, cost/dose includes vaccine price and delivery cost.
• Maternal GBS vaccine is cost-effective at $2 to more than $20/dose, depending on efficacy and disease incidence.
• A maternal GBS vaccine would be cost-effective in low-income sub-Saharan Africa.

## 1 Introduction

Group B streptococcus (GBS) is a leading neonatal sepsis pathogen globally, a major contributor to neonatal deaths in the world’s poorest countries, and has a particularly high burden of disease in sub-Saharan Africa, where half of GAVI-eligible countries are located [1]. In higher-income countries where it has been introduced, intrapartum antibiotic prophylaxis for GBS-colonized women has greatly reduced early-onset GBS (EOGBS) disease, which develops during the first week of life [2]. This strategy, which requires screening cultures of pregnant women several weeks before delivery, availability of screening results at delivery, and the ability to provide intravenous intrapartum antibiotics, may not be feasible in low-income countries. Providing it during delivery to women with clinical risk factors such as intrapartum fever is less complex and costly [3], but less effective and still difficult to implement in resource-poor settings [2]. A trivalent maternal vaccine completed Phase II trials in South Africa and several other countries [4], [5], [6], [7], but further trials were suspended to develop a higher valency vaccine that would cover at least five GBS serotypes (1a, 1b, II, III, and V), which account for almost all cases of infant disease [8]. If a vaccine is successfully developed, antenatal care and/or maternal immunization programs, which already provide tetanus toxoid to women during pregnancy, offer a delivery platform on which to implement maternal GBS immunization, although at additional cost. The vaccine would protect infants not only against EOGBS but also against late-onset disease (LOGBS, which develops between 7 and 90 days).

To speed funders’ decisions about maternal GBS immunization once clinical trials establish efficacy, we evaluated its potential costs and public health impacts (cases prevented, lives saved, disability-adjusted life years [DALYs] averted) in four countries representative of different health and socioeconomic conditions in the 37 GAVI-eligible sub-Saharan countries. We focused on a central policy question – affordable vaccination cost (price plus delivery cost) per dose – and present the highest per-dose costs that would meet two possible cost-effectiveness benchmarks, 0.5 GDP per capita and GDP per capita per DALY averted. In addition, we compare cost-effectiveness ratios to those of other recently introduced new vaccines [9].

## 2 Methods

### 2.1 Analytic overview

The model is structured as a decision tree that describes the two strategies offered to pregnant women, GBS vaccine or no vaccine, with embedded Markov nodes to model the lifetime consequences for their babies, using TreeAge Pro 2016 (TreeAge Inc., Williamstown, MA; see Technical Appendices for details). In the model, pregnant women are subdivided by maternal GBS colonization at delivery (yes/no), then by whether the birth is preterm or term. Babies enter a Markov model (cycle length: 1 year) that simulates pregnancy outcomes (stillbirth, live birth) and the natural history of GBS disease. Only babies born live to colonized mothers are at risk of EOGBS. Although all babies are at risk of LOGBS, the risk is higher among babies born to mothers colonized at the time of delivery. Both EOGBS and LOGBS may present as meningitis or sepsis, which may result in death, long-term disability, or full recovery [10]. An expert panel of published investigators in GBS epidemiology and/or vaccinology, identified through consultation with two authors (SS and JV) and contacted by author AS, provided guidance on model development, parameterization, and analysis; they are listed in the acknowledgments.

We used K-means clustering to group the 37 GAVI-eligible countries into four clusters based on measures of economic development, healthcare infrastructure, and past public health performance [11]. The clusters strike a balance between a region-wide analysis, which averages over a wide range of national circumstances, and country-level analyses, which would show the full range of circumstances but were beyond what the available data and project resources could support. The clusters, defined in the notes to Tables 1 and 2, were robust in a series of sensitivity analysis. Each cluster is represented in the results by the country with median life expectancy.

Table 1 shows, for each example country, the base-case values and ranges of the disease burden parameters in the model. Table 2 shows the base-case values and ranges for the resource and cost parameters. The Technical Appendices provide more information about these parameters.

**Table 1:** Disease burden and efficacy parameters for the sub-Saharan GBS disease prevention cost-effectiveness model.

Variable/Parameter	Base-case value (range) for example country (group #)				Source	Distribution
	Guinea-Bissau (1)	Uganda (2)	Nigeria (3)	Ghana (4)		
Starting age for a Markov node	0					fixed
Constant for age weighting	0					fixed
Discount rate	0.03				[27]; Gates	fixed

*Disease burden*						
Prevalence of maternal colonization	0.218 (0.18–0.26)				[8]	beta
Proportion of births that are preterm	0.11 (0.09–0.14)	0.14 (0.12–0.17)	0.12 (0.11–0.13)	0.15 (0.10–0.18)	[28]	beta
CFR of early onset neonatal GBS meningitis	0.594 (0.40–0.62)	0.283 (0.28–0.56)	0.507 (0.41–0.61)	0.424 (0.25–0.57)	[29], [30] times ratio (2012 NMR example country/Malawi's NMR) from WDI	beta
CFR of late onset neonatal GBS meningitis	0.455 (0.31–0.48)	0.217 (0.22–0.43)	0.388 (0.31–0.47)	0.324 (0.19–0.44)		beta
CFR of early onset GBS sepsis	0.457 (0.31–0.48)	0.218 (0.22–0.43)	0.390 (0.31–0.47)	0.326 (0.19–0.44)		beta
CFR of late onset GBS sepsis	0.289 (0.19–0.30)	0.138 (0.14–0.27)	0.247 (0.20–0.30)	0.206 (0.12–0.28)		beta
EOGBS incidence, per 1000 live births	Reported 1.285 (0.81–1.86); adjusted 3.038 (1.29–4.72)				[8] and technical Appendix A2	beta
LOGBS incidence, per 1000 live births	Reported 0.727 (0.48–1.02); adjusted 1.719 (0.73–2.67)					beta
Relative risk of EOGBS (preterm vs term)	4.123 (0.157–108.24)				meta-analysis and [31], [32]	gamma
Relative risk of LOGBS (preterm vs term)	1.700 (0.854–3.384)					gamma
Relative risk of LOGBS (colonization vs no colonization)	3.050 (1.360–7.180)				[33]	gamma
Rate of stillbirth due to all causes	0.0296 (0.023–0.030)	0.0248 (0.020–0.028)	0.0417 (0.039–0.044)	0.0220 (0.021–0.034)	[34]	beta
Proportion of stillbirths due to GBS	0 (0–0.05)				Expert opinion	beta
Proportion of meningitis among EOGBS cases	0.131 (0.092–0.170)				meta-analysis and [29], [31], [32], [35], [36]	beta
Proportion of meningitis among LOGBS cases	0.528 (0.382–0.673)					beta
Duration of meningitis (days)	17 (14–21)				[37]	uniform
Duration of sepsis (days)	10 (7 −1 4)				[37]	uniform
Proportion of meningitis leading to disabilities	0.440 (0.250–0.650)				[38]	beta
Proportion of sepsis leading to disabilities	0.254 (0.127–0.381)				[39]	beta
Mortality rate, all causes, 2010–2015, by age	Table, Guinea-Bissau	Table, Uganda	Table, Nigeria	Table, Ghana	[40], [41]	fixed
Life expectancy, 2010–2015, by age	Table, Guinea-Bissau	Table, Uganda	Table, Nigeria	Table, Ghana		fixed
Discounted YLL, 2010–2015 by age	Table, Guinea-Bissau	Table, Uganda	Table, Nigeria	Table, Ghana		fixed

*Vaccine effectiveness*						
Proportion of vaccine serotypes among EOGBS	0.974 (0.937–0.996)				[8]	beta
Proportion of vaccine serotypes among LOGBS	0.977 (0.905–1.000)				[8]	beta
Maternal vaccine coverage: ANC1*	0.926 (0.220–0.971)	0.949 (0.743–0.957)	0.606 (0.485–0.727)	0.964 (0.339–0.989)	[42]	beta
Maternal vaccine coverage: ANC4*	0.649 (0.063–0.760)	0.476 (0.442–0.744)	0.510 (0.408–0.612)	0.873 (0.321–0.873)		beta
Vaccine efficacy against covered serotypes, EOGBS	0.50 – 0.90				Expert opinion	beta
Vaccine efficacy against covered serotypes, LOGBS	0.50 – 0.90					beta
Vaccine efficacy adjustment in preterm infants	0.835 (0.779–0.891)				[43] and technical Appendix A4	beta
Vaccine efficacy against maternal colonization	0				Expert opinion	fixed
Vaccine efficacy against preterm	0				Expert opinion	fixed
Vaccine efficacy against stillbirth	0.50–0.90				Expert opinion	beta

**Group 1 (10**): CAR, Guinea, Guinea-Bissau, Mali, Niger, Sierra Leone, Somalia, South Sudan, Chad, DR Congo.

**Group 2 (9)**: Cote d'Ivoire, Cameroon, Lesotho, Mozambique, Mauritania, Sudan, Uganda, Zambia, Zimbabwe.

**Group 3 (1)**: Nigeria.

**Group 4 (17)**: Burundi, Benin, Burkina Faso, Comoros, Eritrea, Ethiopia, Ghana, Gambia, Kenya, Liberia, Madagascar, Malawi, Rwanda, Senegal, Sao Tome/Principe, Togo, Tanzania.

**Table 2:** Cost parameters for the sub-Saharan GBS disease prevention cost-effectiveness model.

Variable/Parameter	Base-case value (range) for example country (group #)				Source	Distribution
	Guinea-Bissau (1)	Uganda (2)	Nigeria (3)	Ghana (4)		
*Health resource use*						uniform
Number of outpatient visits per course of meningitis treatment	3.50 (2.8–4.2)				HRU survey of 13 sub-Saharan experts in care of GBS in infants. Responses were required to be anonymous so resource use by country group could not be identified.	uniform
Number of outpatient visits per course of sepsis treatment	2.42 (1.936–2.904)					uniform
Proportion of neonatal meningitis treated at ICU	0.278 (0.222–0.334)					uniform
Proportion of neonatal sepsis treated at ICU	0.240 (0.192–0.288)					uniform
Proportion of neonatal meningitis treated at paediatric ward	0.722 (0.578–0.866)					uniform
Proportion of neonatal sepsis treated at paediatric ward	0.760 (0.608–0.912)					uniform
Length of stay at ICU, days (meningitis)	8.56 (6.85–10.27)					uniform
Length of stay at ICU, days (sepsis)	6.44 (5.15–7.73)					uniform
Length of stay paediatric ward after ICU discharge, days (meningitis)	4.78 (3.82–5.74)					uniform
Length of stay paediatric ward after ICU discharge, days (sepsis)	3.67 (2.94–4.40)					uniform
Length of stay paediatric ward, days (meningitis)	10.92 (8.74–13.10)					uniform
Length of stay paediatric ward, days (sepsis)	7.50 (6 −9)					uniform

*Unit costs, 2014 US$*						
Cost of an outpatient visit	0.68 (0.54–0.82)	1.43 (1.14–1.72)	23.21 (18.57–27.85)	1.89 (1.51–2.27)	WHO-CHOICE [44] and technical Appendix A5	uniform
Cost of a day in an ICU	2.25 (1.80–2.70)	6.35 (5.08–7.62)	27.73 (22.18–33.28)	8.69 (6.95–10.43)		uniform
Cost of a day on a paediatric ward	2.10 (1.68–2.52)	5.91 (4.73–7.09)	25.83 (20.66–31.00)	8.09 (6.47–9.71)		uniform
Treatment cost for long-term disability	16.32 (13.06–19.58)	34.32 (27.46–41.18)	557.04 (445.68–668.40)	45.36 (36.29–54.43)		uniform
Vaccination cost (price + delivery cost) per dose	7 (2 −1 0)				Technical Appendix A5	

*2010 DALY weights*						
Disability weight for acute meningitis/sepsis	0.210				[45]	fixed
Disability weight for disability due to long-term meningitis/sepsis	0.136					fixed
Number of births, 2013	64,000	1,626,000	7,173,000	800,000	UNICEF	fixed

**Group 1 (10)**: CAR, Guinea, Guinea-Bissau, Mali, Niger, Sierra Leone, Somalia, South Sudan, Chad, DR Congo.

**Group 2 (9)**: Cote d'Ivoire, Cameroon, Lesotho, Mozambique, Mauritania, Sudan, Uganda, Zambia, Zimbabwe.

**Group 3 (1)**: Nigeria.

**Group 4 (17)**: Burundi, Benin, Burkina Faso, Comoros, Eritrea, Ethiopia, Ghana, Gambia, Kenya, Liberia, Madagascar, Malawi, Rwanda, Senegal, Sao Tome/Principe, Togo, Tanzania.

### 2.2 GBS maternal colonization, disease incidence, and serotype distribution

We conducted a systematic review of published literature on the proportion of pregnant women colonized with GBS (maternal carriage); EOGBS and LOGBS disease incidence; and the proportion of GBS disease-causing isolates that would be covered by a pentavalent vaccine (vaccine serotype coverage) in sub-Saharan Africa; we pooled the individual study estimates in a random effects meta-analysis using Open meta-Analyst [http://www.cebm.brown.edu/openmeta/] to estimate overall weighted means and 95% confidence intervals [8]. Since the data did not allow us to differentiate among countries in sub-Saharan Africa, we used the overall means and standard errors for all four of the representative countries.

Reported disease incidence reflects blood culturing practice and its sensitivity as a diagnostic test. We adjusted the estimates of EOGBS and LOGBS disease incidence from the meta-analysis for the proportion of neonates with clinical sepsis undergoing blood culture (90%) and for culture sensitivity (47%) [12], as follows: adjusted incidence = reported incidence/(proportion cultured ∗ culture sensitivity) [3].

### 2.3 GBS case fatality ratios and death from other causes

The only published data on case fatality ratios in sub-Saharan Africa come from a study conducted in Malawi. Case fatality ratios (CFRs) for sepsis and meningitis, by EOGBS and LOGBS, were estimated from that study and adjusted for early versus late onset disease, as well as the underlying risk of neonatal mortality in a country, using methods described in Technical Appendix A3.

Death rates and life expectancies are the 2014 values for the example country from the United Nations’ Population Division [13], [14]. Years of life and disability-adjusted years of life (DALYs) that occurred after the first year of life were discounted at 3%/year.

### 2.4 Maternal GBS vaccination during routine antenatal care

We assumed that GBS vaccine would be delivered to pregnant women in the third trimester and that a single dose would be given for each pregnancy during routine antenatal care. Given the need to administer the vaccine between 27 and 34 weeks of gestation to achieve peak titers in the newborn, the percentage of pregnant women with at least four antenatal visits (ANC4) was used as a proxy for vaccine coverage since women with four visits are likely to attend during the third trimester [15]. In LMICs, however, many pregnant women first attend late in pregnancy and have only 1–2 visits before delivery. Thus ANC1, the percentage of pregnant women with at least one antenatal visit, may be a reasonable alternative proxy for coverage and was used in sensitivity analysis.

### 2.5 Vaccine efficacy

There is no information on the potential efficacy of a pentavalent GBS vaccine. Our expert panel recommended using a range of 50–90%, rather than a single estimate, for serotype-specific vaccine efficacy against EOGBS and LOGBS. Serotype coverage was assumed to be 97.4% for EOGBS and 97.7% for LOGBS, based on the meta-analysis described above [8]. We reduced vaccine efficacy against EOGBS/LOGBS in preterm infants to 0.835 of the efficacy in term infants, using data on the distribution of infants by gestational age and maternal-fetal transfer of antibody; preterm infants were subdivided into those < 34 weeks (6.6% of births) and 34–36 weeks (10.9%), with infants born at 37 weeks or more (82.5%) considered full term (Technical Appendix A4).

### 2.6 *Costs*

Costs were adjusted to 2014 U.S. dollars using the World Bank’s annual GDP deflator series [16] and average annual currency exchange rates [17]. All costs occur during the first year of life, so were not discounted.

*Vaccine price and delivery cost.* In the model we combined price and delivery cost and evaluated *vaccination cost per dose* for the one dose series. In the base-case cost-effectiveness analysis, presented to establish context for the analysis of affordable vaccine costs/dose described below, we used a cost of $7/dose and a range of $2-$10, based on per-dose childhood delivery costs in LMICs [18], and, since no information is available on the likely price of the vaccine in development, UNICEF’s 2016 prices for several multivalent, conjugate vaccines that might serve as reasonable proxies for it (Technical Appendix).

*Treatment costs.* To develop treatment costs we surveyed sub-Saharan experts in GBS disease management to get estimates of the percentages of infants with meningitis and sepsis treated in various settings and the healthcare resources used in those settings. Thirteen of 30 experts responded to the survey. Since their responses were anonymous, we cannot differentiate resource use by cluster. To derive total costs the resource-use estimates were multiplied by WHO-CHOICE unit costs for, as appropriate, an outpatient visit, a bed-day in a paediatric ward, and a bed-day in an intensive care unit, all in secondary-level hospitals [19]. WHO-CHOICE represents only the costs of facilities and personnel, so costs were increased to account for diagnostics, medications, and procedures, assuming treatment cost structures for GBS disease treatment were similar to hospitalized childhood pneumonia in Africa [20].

### 2.7 Cost-effectiveness analysis

Model outputs for maternal GBS immunization and no immunization include EOGBS and LOGBS cases, EOGBS and LOGBS deaths, DALYs, and medical costs. A cost-effectiveness ratio compares two strategies and expresses the comparison as the additional cost of one strategy compared with the other for each additional DALY averted. In this study the cost-effectiveness ratios show the additional cost of maternal GBS immunization, compared with no immunization, for each DALY averted. No age weighting was used in calculating DALYs. One-way sensitivity analysis, in which one model parameter is varied, while holding all other parameters at their base-case values, was conducted to show how the cost-effectiveness of maternal GBS immunization changes as each parameter changes. Results for the most influential parameters were summarized in Tornado diagrams.

Stillbirths account for 2–4% of all births in low-income sub-Saharan countries (Table 1). Preliminary evidence suggests a proportion of stillbirths in sub-Saharan Africa may be caused by GBS [21]. Maternal GBS immunization may prevent some of these deaths. Therefore, we conducted a scenario analysis to explore the potential contribution of preventing GBS-associated stillbirth to the vaccine’s cost-effectiveness.

### 2.8 Calculation of threshold vaccination cost per dose

To estimate the maximum (threshold) affordable vaccination cost/dose in each representative country, we considered two possible cost-effectiveness benchmarks, 0.5 GDP per capita (GDPpc) and GDPpc per DALY averted. Maximum vaccination cost/dose for each representative country was estimated by running a 1-way sensitivity analysis to identify the vaccination cost/dose that produced that benchmark in that country. The sensitivity analysis was repeated for each of three levels of vaccine efficacy and for adjusted and unadjusted disease incidence.

To estimate an uncertainty interval for each threshold vaccination cost/dose we ran a probabilistic sensitivity analysis, holding vaccine efficacy and disease incidence at the levels used to derive the threshold cost/dose, but letting other parameters vary according to the distributions in Table 1, Table 2. A uniform distribution was used for vaccination cost/dose itself, with a lower bound of 50% and an upper bound of 150% of the threshold value. The 5000 PSA iterations were then ranked by their cost-effectiveness ratios and those with cost-effectiveness ratios within 5% of the benchmark were selected. The minimum and maximum vaccination cost/dose associated with those cost-effectiveness ratios provide the bounds of the uncertainty interval around the threshold.

## 3 Results

### 3.1 Health outcomes, costs, and cost-effectiveness

For each representative country Table 3 shows: projected reductions in EOGBS and LOGBS cases, deaths, and DALYs for maternal GBS immunization, compared with no maternal GBS immunization; program costs, treatment costs, and treatment cost savings; and cost-effectiveness ratios. The projections are based on adjusted disease incidence, a vaccine efficacy against covered serotypes of 70%, and vaccination cost/dose of $7. The upper panel shows results for coverage equivalent to ANC4, the lower panel for coverage at ANC1.

**Table 3:** Health outcomes, costs, and cost-effectiveness of maternal GBS immunization in four low-income Sub-Saharan countries, by vaccine coverage.

Maternal vaccine coverage = ANC4	Guinea-Bissau		Uganda		Nigeria		Ghana	
Number of live births	64,000		1,626,000		7,173,000		800,000	
Vaccine is delivered to (number of women)	42,765	64.9%	793,171	47.6%	3,810,778	51.0%	713,765	87.3%
At a program cost of (2014 US$)	$299,358		$5,552,194		$26,675,447		$4,996,354	
And treatment costs of (2014 US$)	$4175		$354,124		$9,193,677		$147,714	
Averting								
EOGBS cases (%)	80	42%	1474	30%	7015	33%	1325	55%
LOGBS cases (%)	47	43%	876	31%	4160	34%	788	57%
EOGBS deaths (%)	38	42%	334	30%	2843	33%	449	55%
LOGBS deaths (%)	18	43%	157	31%	1336	34%	212	57%
DALYs (%)	900	0.10%	9181	0.04%	62,045	0.06%	13,415	0.15%
And saving treatment costs of (2014 US$)	$3051		$156,642		$4,544,167		$188,592	
For a cost/DALY of (2014 US$)	$320		$573		$339		$350	

Maternal vaccine coverage = ANC1	Guinea-Bissau		Uganda		Nigeria		Ghana	

Number of live births	64,000		1,626,000		7,173,000		800,000	
Vaccine is delivered to (number of women)	61,018	92.6%	1,581,342	94.9%	4,528,101	60.6%	788,166	96.4%
At a program cost of (2014 US$)	$427,128		$11,069,396		$31,696,708		$5,517,165	
And treatment costs of (2014 US$)	$2872		$198,470		$8,338,326		$128,056	
Averting								
EOGBS cases (%)	114	59%	2938	60%	8336	38%	1464	61%
LOGBS cases (%)	68	61%	1746	63%	4943	40%	871	63%
EOGBS deaths (%)	54	59%	665	60%	3378	39%	496	61%
LOGBS deaths (%)	25	61%	313	63%	1587	40%	234	63%
DALYs (%)	1284	0.15%	18,309	0.10%	73,738	0.07%	14,808	0.17%
And saving treatment costs of (2014 US$)	$2872		$328,550		$5,399,547		$208,248	
For a cost/DALY of (2014 US$)	$320		$573		$339		$350	

Note: ANC4, the percentage of pregnant women with at least four antenatal visits, and ANC1, the percentage with at least 1 visit, serve as proxies for vaccine coverage. See Section 2.4.

ANC4 varies considerably across the example countries, from 47.6% in Uganda to 87.3% in Ghana (Table 3, upper panel). With maternal GBS immunization coverage at ANC4, cases and deaths prevented range from 30-31% in Uganda to 55–57% in Ghana. Cost per DALY averted is similar for Guinea-Bissau ($320/DALY), Nigeria ($339/DALY), and Ghana ($350/DALY) because the case fatality ratios are similar, and high, in those countries (Table 1). In Uganda, which has the lowest case fatality ratios, there are fewer deaths for GBS immunization to prevent and cost/DALY is $573/DALY.

If the coverage of maternal GBS immunization were ANC1 instead of ANC4, many more cases of disease and death would be prevented – about 60% in Guinea-Bissau, Uganda, and Ghana, all of which have ANC1 rates above 90%. In Nigeria, with ANC1 60.6%, about 40% of cases and deaths would be prevented. Because the percentage of women vaccinated affects vaccination costs, disease treatment costs and cases of disease averted by the same proportion, the cost-effectiveness ratios remain the same whether coverage is equivalent to ANC1 or ANC4, although public health impact increases as more women receive the vaccine.

### 3.2 One-way sensitivity analyses

**Fig. 1:**
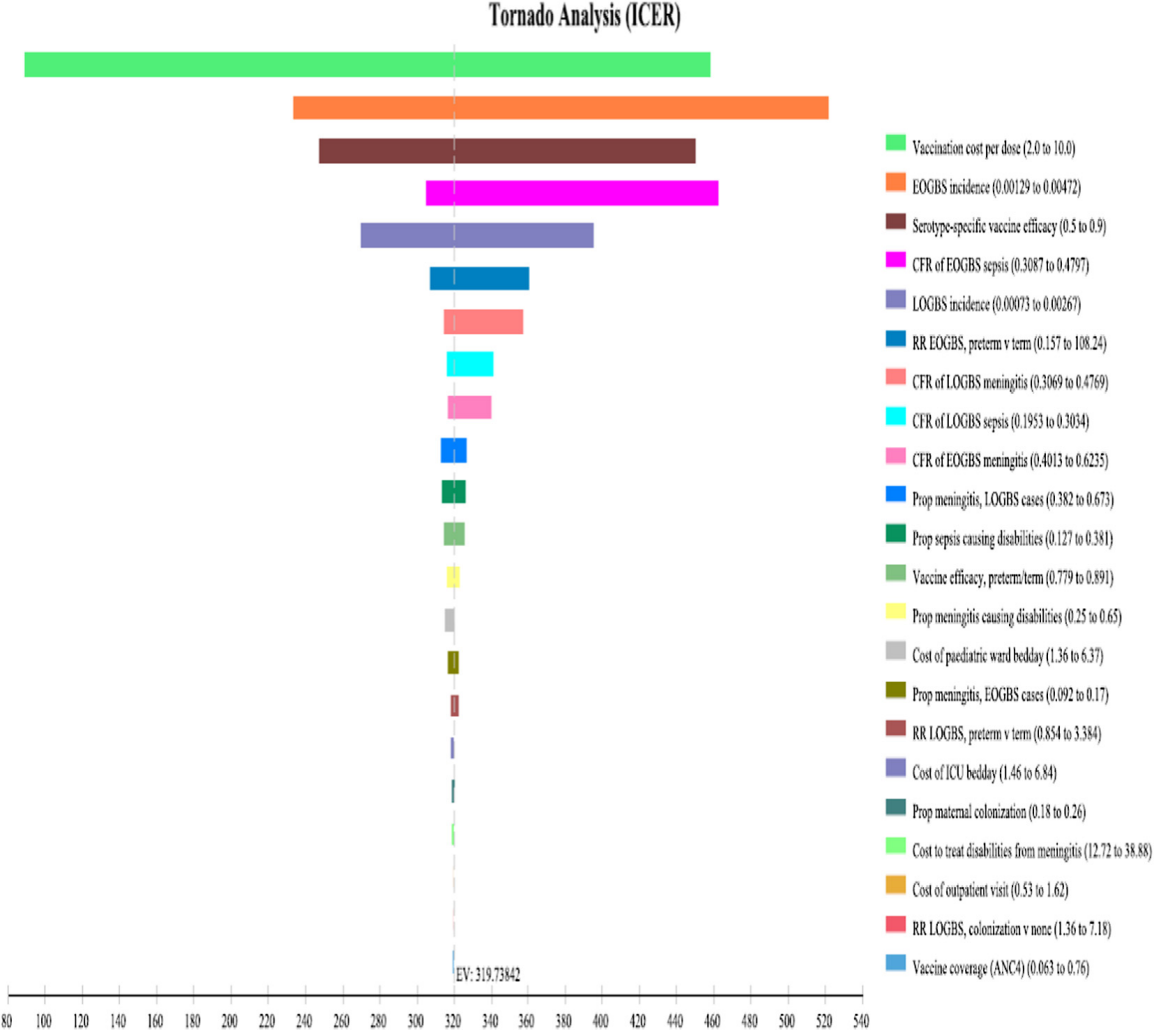
Tornado diagram for Guinea-Bissau. The diagram shows the cost-effectiveness ratio (Cost/DALY) on the horizontal axis with the base-case ratio, $319, indicated by the dashed vertical line. Each horizontal bar shows how Cost/DALY varies around the base-case ratio as that parameter varies across its range (shown in Table 1), while all other parameters are held at their base-case values.

In one-way sensitivity analysis, the same 15 parameters were consistently the most influential in all four countries, so Fig. 1 summarizes the results for those 15 parameters for Guinea-Bissau in the form of a Tornado diagram; Tornado diagrams for Uganda, Nigeria, and Ghana are in Technical Appendix A6. Vaccination cost/dose was consistently the most influential factor. Other influential parameters, in order of declining effect on cost/DALY were the case fatality ratios, vaccine efficacy, LOGBS incidence, and the proportions of cases leading to long-term disability.

### 3.3 Threshold analysis: How much could vaccination cost?

**Fig. 2:**
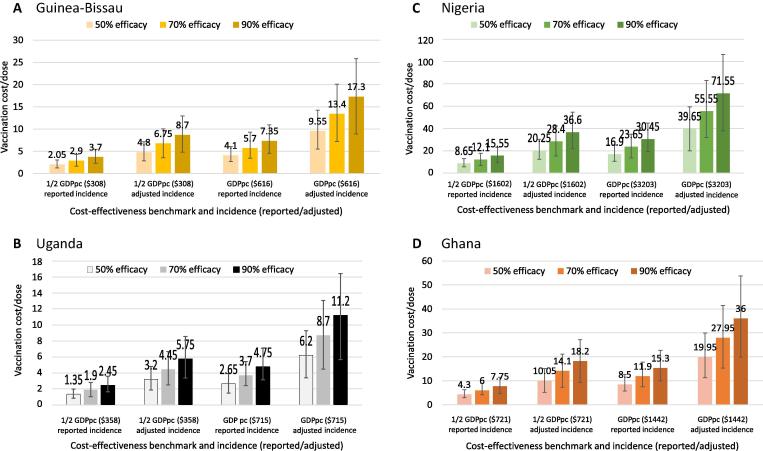
Highest vaccination costs/dose that meet cost-effectiveness benchmarks (and 95% uncertainty intervals), 2014 $.

Fig. 2 shows the maximum (threshold) affordable vaccination cost/dose for each country for two cost-effectiveness benchmarks, 0.5 GDP per capita and GDP per capita per DALY averted, at different levels of disease incidence and vaccine efficacy. Based on reported disease incidence, and assuming 50% serotype-specific vaccine efficacy, for example, vaccination cost/dose in Guinea-Bissau could be, at most, $2.05 to achieve a cost-effectiveness benchmark of $308/DALY averted, half of Guinea-Bissau’s GDP per capita (Fig. 2, Panel A). If the cost-effectiveness benchmark were instead GDP per capita, $616, the vaccination cost/dose could be as much as $4.10. The maximum vaccination cost/dose that meets a given cost-effectiveness benchmark increases if disease incidence is adjusted (higher) and if the vaccine is more effective. For example, if adjusted disease incidence is correct, and the vaccine is 70% effective against covered serotypes, vaccination cost/dose could be as high as $6.75 for the 0.5 GDPpc benchmark or $13.40 for the GDPpc benchmark.

Uganda’s maximum vaccination costs/dose are lower than those of Guinea-Bissau because Uganda has a low neonatal mortality rate, which gives it low GBS CFRs (see Section 2.3). Maximum vaccination cost/dose is $1.35 for reported disease incidence, serotype-specific vaccine efficacy of 50%, and a cost-effectiveness benchmark of 0.5 GDPpc (Fig. 2, Panel B). It rises to $11.20/dose for adjusted incidence, 90% efficacy, and a benchmark of GDPpc.

Maximum vaccination costs/dose are considerably higher in Nigeria, with its higher GDP per capita, ranging from $8.65 to $71.55, depending on disease incidence, vaccine efficacy, and cost-effectiveness benchmark (Fig. 2, Panel C). With a GDPpc intermediate between those of Guinea-Bissau and Uganda on the one hand, and Nigeria on the other, Ghana’s maximum vaccination costs/dose range from $4.30 to $36.00 (Fig. 2, Panel D).

### 3.4 GBS-associated stillbirths

If GBS were associated with 5% of stillbirths (fetal death after 28 weeks [22]), and the vaccine were 70% effective, maternal GBS immunization could prevent many more deaths, perhaps as many as two-thirds more compared with the base-case projections, which assume that GBS is not associated with stillbirth. Such a large increase in DALYs averted, coming at no extra cost since the women would have been vaccinated anyway, could substantially reduce maternal immunization’s cost/DALY. As one example, under the same assumptions as in Table 3, and assuming coverage at ANC4 and 5% of stillbirths caused by GBS, the cost of maternal GBS immunization in Guinea-Bissau would decline from $320/DALY to $168/DALY.

### 3.5 HIV infection

To approximate the cost-effectiveness of maternal GBS immunization for pregnant women with HIV we assumed that the vaccine was only 50% effective and that all four case fatality ratios were at the high end of their ranges for each country. (The two assumptions work in opposite directions: higher death rates mean there are more deaths to prevent, but lower vaccine efficacy means the vaccine is less capable of preventing them.) Under these assumptions, cost/DALY was $430 in Guinea-Bissau; $454 in Uganda; $432 in Nigeria; and $382 in Ghana. Uganda’s cost/DALY went down because the assumed case fatality ratios used were so much higher than those observed in Uganda.

## 4 Discussion

Efficient and affordable interventions are needed to reduce neonatal mortality, especially in parts of the world where it remains high, such as sub-Saharan Africa. Based on a decision analytic model, our analyses suggest that maternal GBS immunization with a pentavalent vaccine that covers most disease-causing GBS serotypes could be cost-effective in low-income sub-Saharan countries. Although the ability to reach large numbers of pregnant women may be constrained by the availability of antenatal care in these countries, substantial numbers of GBS cases and deaths could be prevented because disease burden is high. For example, in Nigeria, 11,000 cases and 4000 deaths (EOGBS and LOGBS) could be averted at a cost of $339 per DALY averted (2014 US$), even if only half of women receive the vaccine (Table 3). Guinea Bissau and Ghana show similar cost-effectiveness ratios. In Uganda the cost is higher, $573/DALY, primarily because the case fatality ratio for GBS cases is relatively low. In all four countries, however, the cost/DALY of maternal GBS immunization is within the range for newer vaccines included in the routine childhood vaccination schedules of these, and other, low-income countries [23].

In threshold analysis, we focused on the range of vaccination costs/dose that would make maternal immunization good value in these countries. Although this study may be most useful for global funders, decision makers, and researchers, recent guidance has emphasized the need for country-driven value criteria [9]. Some studies suggest that 0.5 per capita GDP/DALY may be a reasonable cost-effectiveness threshold for low-income countries [24]. Accordingly, for the threshold analyses, we chose two potential benchmarks: 0.5 GDPpc and GDPpc in each country. If the vaccine is 50% effective against covered serotypes, and if reported disease incidence is correct, we found that affordable vaccination cost/dose ranges from $2-$4 using 0.5 GDPpc as the benchmark. If incidence adjusted for under-reporting is correct rather than reported incidence, vaccination would be cost-effective at a higher cost/dose ($3-$10). If the threshold for cost-effectiveness is per capita GDP, adjusted incidence is correct, and the vaccine is more effective, affordable vaccination cost/dose could exceed $20 for some countries.

One-way sensitivity analysis showed vaccination cost (vaccine price plus delivery cost) and vaccine efficacy, both as yet unknown, to be important determinants of cost-effectiveness. EOGBS and LOGBS disease incidence and case fatality ratios, also important, will lead to variations in the cost-effectiveness of maternal GBS immunization across countries for the same vaccine price and efficacy. Other uncertain factors that were not included in the base case may also be influential. In the base case, for example, we considered only GBS sepsis and meningitis as avertable causes of newborn death and morbidity. GBS may also, however, cause some stillbirths. If these stillbirths were prevented by the vaccine, the DALYs averted would increase substantially, at no extra cost since the women would already have been vaccinated. If as many as 5% of stillbirths are associated with GBS, our analysis shows that the cost/DALY of maternal immunization could drop below $200/DALY.

Our analysis, which considers only immunization costs that vary with the number of women vaccinated, suggests that coverage makes little difference to the cost per DALY averted of maternal GBS immunization. However, coverage is an important determinant of the potential public health impact of the vaccine, its ability to prevent disease and death, as shown by the differences across countries in the percentage of disease averted (Table 3). We used ANC4 as a proxy for vaccine coverage (and ANC1 in sensitivity analysis), a choice supported by the similarity between ANC4 levels and one measure of vaccine coverage, the percentage of women who received two or more doses of tetanus vaccine during pregnancy (TT2). ANC4 may, however, overestimate vaccine coverage. A study of antenatal records in Ghana found, for example, that many pregnant women did not receive the services recommended for a visit [25]. If ANC4 does overstate vaccine coverage, the public health impact of the vaccine will be less than our estimates indicate. When planning for GBS prevention, policymakers will want to consider such differences across and within countries, for example between urban and rural areas. If women in rural areas are less likely than those in urban areas to receive antenatal care, or less likely to receive the vaccine during an antenatal visit, fewer cases and deaths would be averted even if cost/DALY is unchanged.

Our analysis contributes to understanding where future research is most needed. EOGBS and LOGBS incidence and mortality, which are poorly documented in West and Central Africa [8], are key drivers of cost-effectiveness. The role of GBS in stillbirth is also important. Further primary data collection may also be needed about the intra-country distribution of disease (urban versus rural, HIV-infected versus not); the contribution of GBS to preterm delivery; and the contribution of GBS infection in women themselves to GBS disease burden, a topic not considered in our analysis. Further information on the likely program and delivery costs of a maternal GBS vaccine would also help to better understand the vaccine’s value.

The study has several limitations. Firstly, only the variable costs of vaccination were considered. In real-world programs, there may be costs that do not vary with the number of women vaccinated, particularly when a new vaccine is first introduced (e.g. cold chain expansion). In that case cost/DALY would decline as coverage increased and the fixed overhead costs were spread over more women. Secondly, the evidence did not allow us to differentiate disease incidence, a key driver of cost-effectiveness, among countries. We differentiated case fatality ratios by linking them to neonatal mortality, but this approximation may not accurately reflect GBS case fatality. Finally, we assumed that vaccination would not result in herd protection or serotype replacement, because it would not affect gut colonization with GBS, only invasive disease. Other conjugate vaccines, such as pneumococcal vaccines, have led to decreased colonization and hence herd protection, greatly reducing their cost/DALY [26].

## 5 Conclusion

Maternal GBS immunization delivered during antenatal care visits could be a cost-effective public health intervention in low-income sub-Saharan Africa at vaccination costs/dose ranging from $2-$4 to more than $20, depending on disease incidence and vaccine efficacy. The vaccine would be most cost-effective in countries like Nigeria, Guinea Bissau, and Ghana, where the case fatality ratio is high, and less cost-effective in countries like Uganda, where it is relatively low, but its cost/DALY is within the range for newer vaccines already included in the routine childhood vaccination schedules of all these, and other, low-income countries.
