# Supplementary material for: Cost-effectiveness of maternal GBS immunization in low-income sub-Saharan Africa
Source: Vaccine. 2017 Dec 14;35(49Part B):6905–14. doi: 10.1016/j.vaccine.2017.07.108 (PMC5723707; doi:10.1016/j.vaccine.2017.07.108)
Supplement: Supplementary data 1 [file mmc1.docx]

**TECHNICAL APPENDIX**

To accompany “Cost-Effectiveness of Maternal GBS Immunization in low-income Sub-Saharan Africa: Modeling affordable vaccination costs”

Sun-Young Kim, Louise B. Russell, Sri Ram Pentakota, Ben Cosgriff, Anushua Sinha

A report to the Bill & Melinda Gates Foundation

Grant # **OPP1105076**

August 2016

Updated 22 July 2017

**Table of Contents**

Appendix A1: Overview of project and model

Appendix A2: Maternal colonization, EOGBS and LOGBS disease incidence, and serotype distribution

Appendix A3: Case fatality ratios, death from other causes, and life expectancy

Appendix A4: Adjustment of vaccine efficacy in preterm infants

Appendix A5: Costs

Appendix A6: Tornado Diagrams for Uganda, Nigeria, and Ghana

References

**Appendix A1. Overview of Project and Model**

To speed funders’ decisions about maternal GBS immunization, once Phase III trials establish vaccine efficacy, this project was designed to evaluate its potential public health impact (cases and deaths prevented, disability-adjusted life years [DALYs] averted) and costs in the low-income Sub-Saharan region. The evaluation is based on an established projection model, built for an analysis of maternal GBS immunization in South Africa [Kim 2014]. The structure of the model meets the high standards needed for evidence-based policy decisions and was re-fitted with parameter values that represent conditions in low-income Sub-Saharan Africa.

The goal of the project is to help inform decisions about whether, and under what conditions, maternal GBS immunization programs would be a good public health investment in this high burden region. We used the model to make projections for several of the 37 low-income countries in the region and to identify key drivers and decision thresholds for factors, such as vaccine cost per dose, critical to the success of maternal GBS immunization.

**Key questions**

We organized the evaluation around the following key questions:

1. **What are the projected costs and public health benefits** (EOGBS/LOGBS cases and deaths averted and DALYs averted) **of maternal GBS immunization** for GAVI-eligible countries in Sub-Saharan Africa?

2. **What is the projected cost-effectiveness of maternal GBS immunization in this region and how does that depend on vaccine cost per dose?**

3. **What are the key drivers to** which the value of maternal GBS vaccine introduction is sensitive?

4. **What is the value of better information** on key parameters pertaining to disease burden, serotype distribution and prevention effectiveness? Where would investment in further research be most helpful to policy makers?

*The Model*

The projection model, shown in Figure A.1, is structured as a decision tree that describes the interventions offered to pregnant women, with embedded Markov nodes to model the lifetime consequences for their babies. It was built with TreeAge Pro 2012 and updated and evaluated with TreeAge Pro 2015/2016 (TreeAge Inc., Williamstown, MA). In the model, pregnant women are subdivided by maternal GBS colonization at delivery (yes/no), then by whether the birth is preterm or term. Babies enter a Markov model (cycle length: 1 year) that simulates pregnancy outcomes (stillbirth, live birth) and the natural history of GBS disease. Only babies born live to colonized mothers are at risk of EOGBS. LOGBS can be contracted from family or community sources. Both EOGBS and LOGBS may present as meningitis or sepsis, which may result in death, permanent disability or full recovery.

Table 1 in the main paper shows the values and ranges for the disease burden and efficacy parameters used in the model. Table 2 in the main paper shows the values and ranges for the cost parameters.

*Interventions recommended by expert panel.*

*Maternal GBS vaccination during routine antenatal care.* For this strategy we assumed that GBS vaccine would be delivered to pregnant women in the third trimester and that a single dose would be given for each pregnancy. For the base case we assumed that only women who attended at least four antenatal visits (ANC4) would receive the vaccine because four visits indicates that they are likely to attend during the third trimester. ANC4 likely understates the number of women who could be immunized since some women who attend fewer visits start late in pregnancy. ANC1 offers an alternative proxy for coverage but likely overstates the number of women who would receive the vaccine at the appropriate time. Women would receive GBS vaccine in the public sector.

*Maternal GBS vaccination during enriched antenatal care.* Originally, the expert working group for the project proposed that we consider a strategy in which new investments would be made in antenatal care to offer more services to pregnant women in order to induce more of them to attend. The experts suggested the following ways to give pregnant women more incentive to begin and stay with antenatal care: eliminate local co-pays; offer dipstick pregnancy testing, HIV testing, and/or ultrasound; and/or use health extension workers to bring women into care. Global funders have also suggested a range of services that could be used to enhance current antenatal care [Darmstadt 2013].

During our expert panel call on January 28, 2016, the expert working group reviewed the model parameters required by this strategy, the data available to populate them, and the assumptions necessary to perform the analysis. After considering the lack of data linking investments in enriched antenatal care to vaccine uptake, and the lack of information regarding systems’ capability to undertake these investments, the experts changed their recommendation, advising us to limit the evaluation to a comparison of maternal immunization during routine antenatal care and no maternal immunization. They also recommended that we consider risk-factor-based intrapartum antibiotic prophylaxis (RFB-IAP) in scenario analysis, not because they considered it a practical option in these countries but because it would come up during discussions of maternal GBS immunization and it would be helpful to have some information about its likely impact.

*How Countries Were Grouped*

A regional analysis was originally proposed, but to match more closely the range of conditions facing the 37 GAVI-eligible Sub-Saharan countries we decided instead to conduct the evaluation for subgroups of countries. We developed four country groups using a statistical grouping method, cluster analysis, subject to expert review. We used the most common statistical clustering analysis algorithm, K-means, and the most common measure of distance for statistical clustering, Euclidean distance, to group the 37 countries on the basis of 24 readily available measures of economic development, general health resources, and past success in public health programs. Two, three, and four groups were explored, tested for robustness, and reviewed by our expert panel. Public health performance was consistently important in determining the groups. The details of the analysis and the expert review are described in more detail in Russell 2017.

The experts preferred four groups to fewer, on the ground that national decision makers would more readily recognize their own country among the four groups. The countries in each group are shown in the notes to Tables 1 and 2 in the main paper; Nigeria consistently fell into a group by itself. To make it easier to interpret the findings we decided to show results for one country in each group and chose as the example country the one with the median life expectancy for its group: Guinea-Bissau (Group 1); Uganda (Group 2); Nigeria (the only country in Group 3); and Ghana (Group 4).

*Cost-effectiveness analysis*

Projected outputs for maternal GBS immunization and no immunization include EOGBS and LOGBS cases, EOGBS and LOGBS deaths, disability-adjusted life-years (DALYs), and medical costs. A cost-effectiveness ratio compares two strategies, in this case maternal immunization and no immunization, and expresses the comparison as the additional cost of the more expensive strategy for each additional DALY averted. Health outcomes and costs were discounted at 3% per year. No age-weighting was used in the estimation of DALYs.

*Calculation of Threshold Vaccine Prices*

Since GBS vaccine is still under development and its price is not known we focused on calculating the maximum per-dose vaccination cost (price plus delivery cost) that would make maternal GBS immunization cost-effective at two cost-effectiveness benchmarks: ½ GDP per capita per DALY averted; and GDP per capita per DALY averted. GDP per capita/DALY has been recommended by the World Health Organization, although that recommendation is under review [Marseille 2015]. Half of GDP per capita/DALY has been suggested as perhaps more appropriate in low-income countries, to prevent new services displacing existing services that contribute more to good health [Woods 2015].

For each cost-effectiveness benchmark, the per-dose vaccination cost that produced that benchmark was estimated by running a 1-way sensitivity analysis. For example, GDP per capita, in 2014 US dollars, was $616 in Guinea-Bissau, so the 1-way sensitivity analyses identified the per-dose vaccination cost that yielded a DALY for $308 (1/2 GDP per capita) and $616. Per-dose vaccination cost was estimated separately for two levels of disease incidence, reported and after adjustment for underreporting, and three levels of vaccine efficacy against covered serotypes, 50%, 70%, and 90% (as recommended by our expert panel), for a total of six estimates for each benchmark. All other parameters were held at their base-case values, shown in Tables 1 and 2 in the main paper.

To estimate an uncertainty interval for each vaccination cost threshold we ran a probabilistic sensitivity analysis (PSA), holding disease incidence and vaccine efficacy at the values used to derive the threshold, but letting other parameters vary according to the distributions in Tables 1 and 2. A uniform distribution was used for per-dose vaccination cost itself, with a lower bound of 50% and an upper bound of 150% of the threshold cost. The PSA results were then ranked by their cost-effectiveness ratios and those with cost-effectiveness ratios within 5% of the benchmark, e.g., $293-$323 for a benchmark of $308/DALY, were selected. The minimum and maximum vaccination costs associated with those cost-effectiveness ratios provide the bounds of the uncertainty interval around the projected per-dose vaccination cost.

**Figure A1.1.** Diagram of the GBS disease prevention model. Purple circles indicate embedded Markov nodes


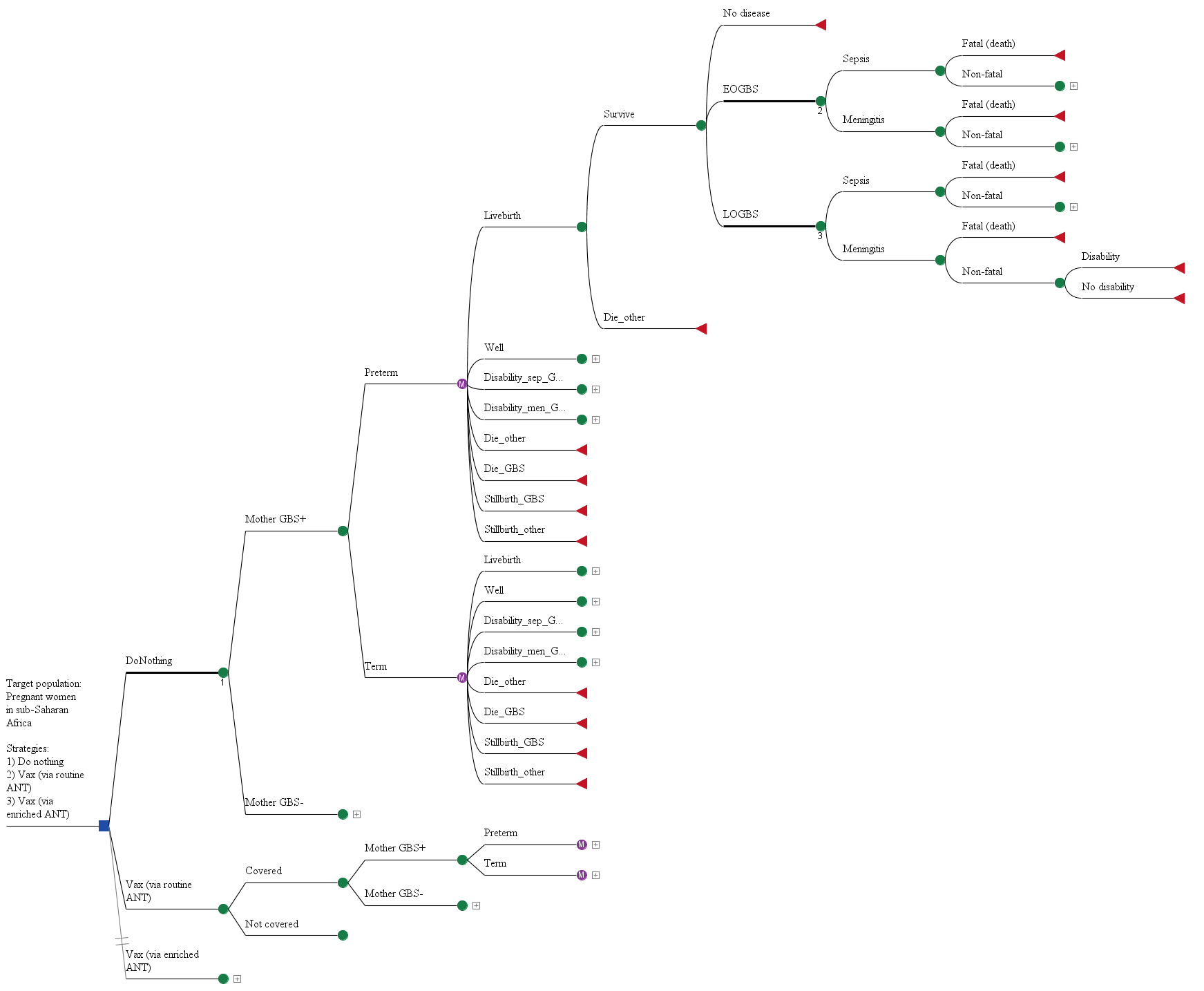


**Appendix A2: Maternal colonization, EOGBS and LOGBS disease incidence, and serotype distribution**

We conducted a systematic review of the published literature on the proportion of pregnant women colonized with GBS (maternal carriage), EOGBS and LOGBS disease incidence, and the proportion of GBS disease-causing isolates that would be covered by a pentavalent vaccine (vaccine serotype coverage) in Sub-Saharan Africa [Sinha 2016]. We pooled the individual study estimates in a random effects meta-analysis using Open Meta-Analyst [http://www.cebm.brown.edu/openmeta/] to estimate the overall weighted means and 95% confidence intervals shown in Table 1 in the main paper. See Sinha 2016 for more details.

Reported disease incidence is an underestimate of true incidence because not all infants are cultured for GBS and the blood culture is not sensitive enough to identify all GBS cases. Using data from centers in South Africa [Kellogg 1997], we adjusted the pooled estimates of EOGBS and LOGBS disease incidence from the systematic literature review for the proportion of neonates with clinical sepsis who were cultured (90%) and culture sensitivity (47%) as follows:

adjusted incidence = reported incidence/(proportion cultured * culture sensitivity)

adjusted incidence = reported incidence/(0.90 * 0.47)

adjusted incidence = 2.364 * reported incidence

We present results for both reported and adjusted disease incidence in the final report.

The ranges for reported incidence came from the systematic literature review. We calculated the upper bounds of the ranges for adjusted disease incidence by multiplying the reported values by the ratio of probable EOGBS to culture-confirmed EOGBS, 3.67, from a recent South African trial [Cutland 2009]. The lower bounds are the reported estimates from the meta-analysis.

**Appendix A3. Case fatality ratios, death from other causes, and life expectancy**

*GBS Case fatality ratios and death from other causes*

Four case fatality ratios (CFRs) were estimated, one each for EOGBS meningitis, LOGBS meningitis, EOGBS sepsis, and LOGBS sepsis, using data from a study of neonatal sepsis in Malawi [Milledge 2005] and relative risks from a U.S. study [Schrag 2000]. The details of the calculations are explained in the next section.

Since GBS is one cause of neonatal mortality we reasoned that death rates from GBS would be higher in countries with higher neonatal mortality. We derived CFRs for each country by multiplying the four Malawian ratios by the ratio of the neonatal mortality rate (NMR) in the country to the NMR in Malawi. We used the adjusted CFRs for the example country as the base-case values for the country group and the ranges across countries in the group as the ranges for sensitivity analysis (see Table 1 in the main paper). NMRs for 2012 were taken from the World Bank’s World Development Indicators (WDI) web site [World Bank b].

Death from other causes during the first year was set at the infant mortality rate (IMR), also from the WDI. Because GBS contributes a small proportion to the total, we did not adjust the reported IMRs for GBS. The mortality rates and life expectancies by age, used to project life expectancy over an infant’s lifetime, are from the United Nations Population Division [UNa, UNb]. Data for the example country were used for the base case and were not varied in sensitivity analysis.

*Calculation of the Malawian Case Fatality Ratios*

The Malawi study [Milledge 2005] presented GBS deaths and cases, stratified by meningitis versus bacteremia/sepsis, and, separately, by overall early-onset (EOGBS) versus late-onset (LOGBS), but it did not stratify GBS meningitis into early- versus late-onset or GBS sepsis into early- versus late-onset. Relative risks (RRs) for early- versus late- onset disease were derived from [Schrag 2000] to further stratify the Malawi into (1) early-onset meningitis, (2) late-onset meningitis, (3) early-onset sepsis, and (4) late-onset sepsis.

As an example, consider the calculation for two unknowns, early-onset and late-onset GBS meningitis cases. The Malawi study described the overall number of GBS meningitis cases, the number of all EOGBS (any syndrome), and the number of all LOGBS (any syndrome). From the Schrag study, we have the relative risk of early versus late onset meningitis, where this RR is defined as (early-onset meningitis/ all EOGBS)/ (late-onset meningitis/ all LOGBS). We can then write the following system of two equations, derived from a 2x2 table analysis, and solve for the two unknowns, # EO meningitis cases and # LO meningitis cases. The RR is from the Schrag study; the other known values are from the Malawi study.

**Eq. 1** (# EO meningitis cases) = (# LO meningitis cases) x RR x (# all EOGBS cases)/ (# all LOGBS cases)

**Eq. 2 (**# EO meningitis cases) = (# all GBS meningitis cases) - (# LO meningitis cases)

Analogous calculations were performed to estimate the numbers of early-onset versus late-onset GBS bacteremia/sepsis cases, and the numbers of deaths for each of the four categories. Four case fatality ratios for Malawi were then estimated by dividing estimated deaths by cases, for into early-onset meningitis, late-onset meningitis, early-onset bacteremia/sepsis, and (4) late-onset bacteremia/sepsis..

Table A3.1 Information used to estimate GBS case fatality ratios

| **Item** | **Value** |
| --- | --- |
| *Malawi study* | |
| Number of early-onset GBS cases | 61 |
| Number of late-onset GBS cases | 75 |
| Number of GBS meningitis cases | 60 |
| Number of GBS bacteremia/sepsis cases | 76 |
| Number of GBS meningitis deaths | 16 |
| Number of GBS bacteremia/sepsis deaths | 16 |
| *Schrag study* | |
| RR, early versus late-onset GBS meningitis | 0.23 |
| RR, early- versus late-onset GBS bacteremia/sepsis | 1.27 |
| RR, early-versus late-onset GBS disease death | 1.44 |
| *2015 Neonatal mortality rates (per 1,000 births)* | |
| Malawi | 21.8 |
| Cluster 1 | 38.6 (26.8 – 42.6) |
| Cluster 2 | 27.1 (18.7 – 37.9) |
| Cluster 3 | 34.3 |
| Cluster 4 | 24.1 (17.1 – 34.0) |

**Appendix A4. Adjustment of vaccine efficacy in preterm infants**

*Vaccine efficacy*

The protection conferred by maternal immunization may result from passive transfer of maternal GBS antibody to the infant, reduced maternal carriage of vaccine serotypes, or both. There is no information on the efficacy of a pentavalent GBS vaccine. Our expert panel recommended using a range of 50%-90% for serotype-specific vaccine efficacy against EOGBS and LOGBS, rather than a single estimate.

Because transfer of maternal antibody depends on gestational age, we adjusted vaccine efficacy downward for infants delivered before 37 weeks. Table A4.1 summarizes the data and calculations used to arrive at the adjustment of vaccine efficacy for a South African analysis of maternal GBS immunization [Lin 2004; Kim 2014]. Using the same approach, the proportions of births that were preterm in the 37 Sub-Saharan countries in 2010 [WHOc], and estimates of how those proportions were distributed between <34 weeks and 34-36 weeks, we calculated an average adjustment factor of 0.835 for this analysis. To derive a standard error for this estimate, we treated the 169 infants (29 cases, 143 controls) in Lin’s 2004 study as a random sample, and applied the formula for the standard error of a proportion – the square root of [(p*(1-p))/N], where p=0.835 and N=169 – which produced a standard error of 0.0286 and a 95% confidence interval for sensitivity analysis of 0.779-0.891.

Table A4.1. Adjustment of vaccine efficacy (VE) for preterm delivery in **South Africa**. *Unshaded cells are the data, shaded cells the calculations based on those data.*

| Gestational age | Maternal-fetal transfer of antibody, % of maternal level [Lin 2004] | Vaccine efficacy (VE) relative to term delivery | Gestational age distribution, South Africa | Fraction of all preterm deliveries | Weighted average of VE for preterm delivery |
| --- | --- | --- | --- | --- | --- |
| <34 weeks | 56% | ~73% (=56/77) of VE for term delivery | 6.6% | 37.7% (=6.6/17.5) | **81%** (=73%*0.377+  86%*0.623)  **of VE for term delivery** |
| 34-36 weeks | 66% | ~86% (=66/77) of VE for term delivery | 10.9% | 62.3% (=10.9/17.5) |  |
| ≥ 37 weeks | 77% | 1 (=77/77) | 82.5% | -- | -- |

**Appendix A5. Costs**

Costs were estimated from the perspective of the healthcare system and include the costs of maternal GBS immunization, EOGBS and LOGBS disease treatment, and long-term disability from EOGBS/LOGBS. All costs were adjusted for inflation using the World Bank’s GDP deflator series [World Bank a] and were converted to 2014 US dollars using average annual currency exchange rates from the International Monetary Fund [IMF]. The base-case cost is the cost in the example country; the range used in sensitivity analysis is, unless stated otherwise, +/-20% of the base-case value.

*Coverage*

Since maternal GBS vaccine would be offered during pregnancy, as part of antenatal care, we used data on antenatal care visits from WHO [WHOb] as a proxy for maternal GBS vaccine coverage. The proportion of pregnant women with at least four antenatal visits (ANC4) was used for the base case, since it is very likely that these women would attend during the third trimester when the vaccine should be given. The proportion of women with at least one antenatal visit (ANC1) is an alternative measure of vaccine coverage since many women in low-income countries have only one antenatal visit and that one often occurs in the third trimester. For each measure, the range is the range for countries in the country group, except for Nigeria for which the range is +/-20% of the reported value.

*Vaccine price and delivery cost.*

Because the GBS vaccine is still under development, no market price is available nor is there information on its presentation. For the base case, on the advice of our expert panel, we assumed that GBS vaccine would be provided in the form of a pre-filled syringe. The following table of multivalent vaccine products suggests the range of prices at which maternal GBS vaccine might be offered. **Table A5.1. UNICEF prices for multivalent vaccines**

| UNICEF prices (2016 US $) | | | Estimated cost of goods (Price less 15%) | | |
| --- | --- | --- | --- | --- | --- |
| Vaccine | Doses/vial | Price/dose | Low | Base | High |
| HPV 2-valent | 2 dose | 4.60 | 3.13 | 3.91 | 4.69 |
| HPV 4-valent | 1 dose | 4.50 | 3.06 | 3.83 | 4.59 |
| PCV 13-valent | 1 dose | 3.50 | 2.38 | 2.98 | 3.57 |
| PCV 10-valent | 2 dose | 3.50 | 2.38 | 2.98 | 3.57 |
| MenA 1-valent | 10 dose | 0.64 | 0.44 | 0.55 | 0.66 |

We based our analysis on *total variable vaccination cost per dose*, which combines the per-dose price of the vaccine itself and the variable costs of delivering a dose to a pregnant woman. Variable delivery costs, that is, costs that vary with the number of women vaccinated, include some program activities; distribution, transport, cold chain, safety boxes, and wastage costs for the vaccine; personnel at the delivery site; and treatment of adverse vaccination events.

For the conventional cost-effectiveness analysis, we combined the vaccine prices per dose in Table A5.1 with the per-person delivery costs estimated for infant pertussis vaccine [Russell 2016] as an approximation of the delivery costs of maternal GBS vaccine. The sum ranged from about $2 to about $10, so we used $7 for the base case and $2-$10 for sensitivity analysis. In addition, for two cost-effectiveness benchmarks for each example country, ½ GDP per capita/DALY and GDP per capita/DALY, we estimated the highest vaccination cost per dose that would meet the benchmark. Decision makers can deduct the cost of delivery in a given country to determine the maximum price the country could pay for the vaccine and still meet the cost-effectiveness benchmark.

*Disease treatment costs.*

As a basis for treatment costs, in November-December 2014 we conducted a survey of Sub-Saharan experts in GBS disease and its management. Thirteen of 30 experts, identified with the help of our expert panel, responded to the survey. Since the Institutional Review Board required that their responses be kept anonymous, we could not identify the country or the respondent and so cannot differentiate resource use by country or country group. The survey supplied estimates of the percentages of infants with meningitis and infants with sepsis treated in various settings (intensive care unit, pediatric ward, or both, and outpatient visits) in low-income Sub-Saharan Africa, the length of stay or number of visits in each setting for each syndrome, and some of the diagnostic and therapeutic resources used in those settings (Table 2 in the main paper). In the survey we asked separately about stays in pediatric and short-stay wards, but the responses indicated that the two were treated as the same thing, with the location of answers, pediatric or short-stay, based on the terminology most common in the respondent’s country; thus we combined pediatric and short-stay information and use the term “pediatric ward” for the combined information in Table 2. We used the means of the responses for the base case.

To derive treatment costs we multiplied the resource estimates from the survey by appropriate unit costs from WHO [WHO-CHOICE]. For inpatient stays, we used bed-day costs in intensive care units and pediatric wards at secondary-level hospitals, converting 2008 costs in local currency to 2014 US dollars as described in the introduction to this appendix. For outpatient visits we used the unit cost for an outpatient visit to a secondary-level facility.

WHO-CHOICE unit costs represent facility and personnel costs, but do not include the costs of diagnostic tests, medications, or procedures. To add these costs we used mark-ups derived from a study of acute lower respiratory tract infection in South African children under 5 years of age [Sinha 2012]. The mark-ups applied were:

1. Intensive care unit, 31.41% (the sum of 9.07% for diagnostic tests, 8.28% for medications, and 14.06% for procedures);
2. Pediatric ward, 22.4% (the sum of 16.77% for diagnostic tests, 5.14% for medications, and 0.49% for procedures);
3. Short-stay ward (which was combined with pediatric ward, as described above), 13.38% (the sum of 8.62% for diagnostic tests, 4.76% for medications, and 0% for procedures);
4. Sinha 2012 did not report data for outpatient visits, so we increased the WHO-CHOICE unit costs by 10%.

*Long-term disability costs*

To approximate the costs of treating long-term disabilities from GBS disease, we conservatively assumed monthly outpatient visits for two years after hospital discharge, following the approach of a previous study in South Africa [Kim 2014].

**Appendix 6. Tornado Diagrams for Uganda, Nigeria, and Ghana**

A Tornado diagram shows a series of 1-way sensitivity analyses. A 1-way sensitivity analysis varies one model parameter, while holding all other parameters at their base-case values, to calculate how the cost-effectiveness of maternal GBS immunization changes, compared with no immunization, as the parameter changes. The range for each parameter is shown in Tables 1 and 2 of the main paper and in the diagram legend.

The cost-effectiveness ratio appears on the horizontal axis of the diagram. The vertical line running approximately up the middle of the diagram shows the cost-effectiveness ratio when all parameters take their base-case values. Each horizontal bar represents the 1-way sensitivity analysis for one parameter and shows how the cost-effectiveness ratio changes as that parameter changes. The parameters are shown in order of influence, starting from the top, hence the name “Tornado” diagram. The parameter is identified by color of the bar, which is also shown next to the name of the parameter in the legend to the right.

A Tornado diagram for Guinea-Bissau was included in the main text of the final report. Because the diagrams for all four countries showed the same factors to be most important, we relegated the other three Tornado diagrams to this appendix. The diagrams on the next three pages show, as did the diagram for Guinea-Bissau, 1-way sensitivity analyses for the 15 parameters that had the biggest influence on the cost-effectiveness of maternal GBS immunization.

**UGANDA**

**NIGERIA**

**GHANA**

References

Berger MB, Xu X, Williams JA, Van de Ven CJ, Mozurkewich EL. Early hospital discharge of infants born to group B streptococci-positive mothers: a decision analysis. BJOG. 2012 Mar;119(4):439-48. doi: 10.1111/j.1471-0528.2011.03249.x. Epub 2012 Jan 18.

Bomela HN, Ballot DE, Cooper PA. Is prophylaxis of early-onset group B streptococcal disease appropriate for South Africa? SAMJ, S. 2001;91(10):858-60.

Cutland CL, Madhi SA, Zell ER, Kuwanda L, Laque M, et al. Chlorhexidine maternal-vaginal and neonate body wipes in sepsis and vertical transmission of pathogenic bacteria in South Africa: a randomised, controlled trial. Lancet 2009; 374: 1909-1916.

Darmstadt GL, Marchant T, Claeson M, et al. A strategy for reducing maternal and newborn deaths by 2015 and beyond. BMC Pregnancy Childbirth. 2013 Nov 22;13:216. doi: 10.1186/1471-2393-13-216.

Gray KJ, Bennett SL, French N, Phiri AJ, Graham SM. Invasive group B streptococcal infection in infants, Malawi. Emerg Infect Dis. 2007;13(2):223-9.

Haffejee IE, Bhana RH, Coovadia YM, Hoosen AA, Marajh AV, Gouws E. Neonatal group B streptococcal infections in Indian (Asian) babies in South Africa. Journal of Infection. 1991;22(3):225-31.

HRU (Health Resources Utilization Survey). Survey of 13 experts in Sub-Saharan GBS disease and management conducted November-December 2014 by the Rutgers research team.

International Monetary Fund (IMF), International Financial Statistics. World Development Indicators. Official exchange rate (LCU per US$, period average). Last Updated12/22/2015. At: [http://data.worldbank.org/indicator/PA.NUS.FCRF. Last accessed March 2016](http://data.worldbank.org/indicator/PA.NUS.FCRF.%20Last%20accessed%20March%202016).

Kellogg JA, Ferrentino FL, Goodstein MH, Liss J, Shapiro SL, et al. Frequency of low level bacteremia in infants from birth to two months of age. Pediatr Infect Dis J 1997;16: 381-385.

Kim SY, Russell LB, Park J, Verani JR, Madhi SA, Cutland CL, Schrag SJ, Sinha A. Cost-effectiveness of a potential group B streptococcal vaccine program for pregnant women in South Africa. Vaccine. 2014 Apr 7;32(17):1954-63. doi: 10.1016/j.vaccine.2014.01.062. Epub 2014 Feb 11.

Lin F-YC, Weisman LE, Troendle J, Adams K. Prematurity is the major risk factor for late-onset group B streptococcus disease. J Infect Dis 2003;188: 267-271.

Lin FY, Weisman LE, Azimi PH, Philips JB, 3rd, Clark P, et al. Level of maternal IgG anti-group B streptococcus type III antibody correlated with protection of neonates against early-onset disease caused by this pathogen. J Infect Dis 2004;190: 928-934

Libster R, Edwards KM, Levent F, Edwards MS, Rench MA, et al. Long-term outcomes of group B streptococcal meningitis. Pediatrics 2012;130: e8-15

Madhi SA, Radebe K, Crewe-Brown H, Frasch CE, Arakere G, [Mokhachane M](http://www.ncbi.nlm.nih.gov/pubmed/?term=Mokhachane%20M%5BAuthor%5D&cauthor=true&cauthor_uid=12648320), [Kimura A](http://www.ncbi.nlm.nih.gov/pubmed/?term=Kimura%20A%5BAuthor%5D&cauthor=true&cauthor_uid=12648320). (2003) High burden of invasive Streptococcus agalactiae disease in South African infants. Ann Trop Paediatr 23: 15-23.

Marseille E, Larson B, Kazi DS, Kahn JG, Rosen S. Thresholds for the cost-effectiveness of interventions: alternative approaches. Bull World Health Organ, 2015; 93:118-124.

Milledge J, Calis JC, Graham SM, Phiri A, Wilson LK, Soko D, et al. Aetiology of neonatal sepsis in Blantyre, Malawi: 1996-2001. Ann Trop Paediatr. 2005;25(2):101-10.

Russell LB, Bhanot G, Kim S-Y, Sinha A. Using Cluster Analysis to Group Countries for Cost-Effectiveness Analysis: An Application to Sub-Saharan Africa. Medical Decision Making, in press.

Russell LB, SR Pentakota, CM Toscano, B Cosgriff, A Sinha. What pertussis mortality rates make maternal aP immunization cost-effective in low- and middle-income countries? A Decision Analysis. Clin Infect Dis, in review.

Salomon JA, Vos T, Hogan DR, Gagnon M, Naghavi M, et al. Common values in assessing health outcomes from disease and injury: disability weights measurement study for the Global Burden of Disease Study 2010. Lancet 2012;380: 2129-2143.

Schrag SJ, Cutland CL, Zell ER, Kuwanda L, Buchmann EJ, et al. Risk factors for neonatal sepsis and perinatal death among infants enrolled in the prevention of perinatal sepsis trial, Soweto, South Africa. Pediatr Infect Dis J 2012;31: 821-826.

Schrag, S.J., et al., Group B streptococcal disease in the era of intrapartum antibiotic prophylaxis. N Engl J Med, 2000. 342(1): p. 15-20.

Simonsen KA, Anderson-Berry AL, Delair SF, Davies HD. Early-onset neonatal sepsis. Review. Clin Microbiol Rev, 2014 Jan. 27(1): p. 21-47.

Sinha A, Kim S, Ginsberg G, Franklin H, Kohberger R, Strutton D, Madhi SA, Griffiths UK, Klugman KP. Economic burden of acute lower respiratory tract infection in South African children. Paediatr Int Child Health. 2012;32(2):65-73

Sinha A, Russell LB, Tomczyk S, Verani JR, Schrag SJ, Berkley JA, Mohammed M, Sigauque B, Kim SY; GBS Vaccine Cost-Effectiveness Analysis in Sub-Saharan Africa Working Group. [Disease Burden of Group B Streptococcus among Infants in Sub-Saharan Africa: A Systematic Literature Review and Meta-Analysis.](http://www.ncbi.nlm.nih.gov/pubmed/27213263) Pediatr Infect Dis J. 2016 May 20. [Epub ahead of print]

Sivanandan S, Soraisham AS, Swarnam K. [Choice and duration of antimicrobial therapy for neonatal sepsis and meningitis.](http://www.ncbi.nlm.nih.gov/pubmed/22164179) Int J Pediatr. 2011. 2011; article 712150. doi: 10.1155/2011/712150. Epub 2011 Nov 20.

United Nations, Population Division (UNa). File MORT/17-1: Abridged life table, for both sexes combined, by major area, region and country, 1950-2100 Estimates, 1950 – 2015. At <https://esa.un.org/unpd/wpp/Download/Standard/Population/> Accessed July 2016.

United Nations, Population Division (UNb). File MORT/16-1: Life expectancy at exact age, e(x), for both sexes combined, by major area, region and country, 1950-2100 Estimates, 1950 – 2015. At <https://esa.un.org/unpd/wpp/Download/Standard/Population/> Accessed July 2016.

UNICEF. The State of the World's Children Report 2015 Statistical Tables - Tables by thematic area. <http://www.data.unicef.org/resources/the-state-of-the-world-s-children-report-2015-statistical-tables.html> Accessed July 2016.

Woods B, Revill P, Sculpher M, Claxton K. Country-level cost-effectiveness thresholds: initial estimates and the need for further research. CHE Research Paper 109. March 2015. Centre for Health Economics, University of York. Available at www.york.ac.uk/che/publications/.

World Bank national accounts data, and OECD National Accounts data files (World Bank a). World Development Indicators. Inflation, GDP deflator (annual %). Last Updated12/22/2015. At: <http://data.worldbank.org/indicator/NY.GDP.DEFL.KD.ZG> Last accessed March 2016.

World Bank, World Development Indicators (World Bank b). Mortality rate, neonatal (per 1,000 live births) <http://data.worldbank.org/indicator/SH.DYN.NMRT> Last accessed July 2016. Substitute UN or perhaps WHO GHO? <http://apps.who.int/gho/data/view.main.CM1320R>

World Health Organization (WHOa). Country stillbirth rates per 1000 total births for 2009. At <http://www.who.int/pmnch/media/news/2011/stillbirths_countryrates.pdf> Last accessed July 2016.

World Health Organization, Choosing Interventions that are Cost Effective (WHO-CHOICE). Country-specific Unit Costs. At: <http://www.who.int/choice/country/country_specific/en/> Last accessed September 2016.

World Health Organization, Global Health Observatory (WHOb). Antenatal care coverage, data by country. At <http://apps.who.int/gho/data/view.main.321> Last accessed July 2016.

World Health Organization, Global Health Observatory (WHOc). Infant nutrition, data by country (preterm birth rate 2010). At <http://apps.who.int/gho/data/view.main.1730> Last accessed July 2016.
